# Phytochemical Profile, Antioxidant Potential and Toxicity Evaluation of the Essential Oils from *Duguetia* and *Xylopia* Species (*Annonaceae*) from the Brazilian Amazon

**DOI:** 10.3390/antiox11091709

**Published:** 2022-08-30

**Authors:** Márcia Moraes Cascaes, Ângelo Antônio Barbosa De Moraes, Jorddy Neves Cruz, Celeste de Jesus Pereira Franco, Renan Campos E Silva, Lidiane Diniz do Nascimento, Oberdan Oliveira Ferreira, Tainá Oliveira dos Anjos, Mozaniel Santana de Oliveira, Giselle Maria Skelding Pinheiro Guilhon, Eloisa Helena de Aguiar Andrade

**Affiliations:** 1 Programa de Pós-Graduação em Química, Universidade Federal do Pará, Rua Augusto Corrêa S/N, Guamá, Belém 66075-900, PA, Brazil; 2 Laboratório Adolpho Ducke-Coordenação de Botânica, Museu Paraense Emílio Goeldi, Av. Perimetral, 1901, Terra Firme, Belém 66077-830, PA, Brazil; 3 Laboratory of Functional and Structural Biology, Institute of Biological Sciences, Universidade Federal do Pará, Rua Augusto Corrêa S/N, Guamá, Belém 66075-900, PA, Brazil; 4 Programa de Pós-Graduação em Biodiversidade e Biotecnologia-Rede Bionorte, Universidade Federal do Pará, Rua Augusto Corrêa S/N, Guamá, Belém 66075-900, PA, Brazil; 5 Programa de Pós-Graduação em Ciências Biológicas-Botânica Tropical, Museu Paraense Emilio Goeldi/Universidade Federal Rural da Amazônia, Av. Perimetral, 1901, Terra Firme, Belém 66077-830, PA, Brazil

**Keywords:** natural products, Amazon, medicinal plant, volatile compounds, bioactive compounds

## Abstract

The essential oils (EOs) of Duguetia echinophora, D. riparia, Xylopia emarginata and X. frutescens (Annonaceae) were obtained by hydrodistillation and the chemical composition was analyzed by GC-MS. An antioxidant assay using the ABTS and DPPH radicals scavenging method and cytotoxic assays against Artemia salina were also performed. We evaluated the interaction of the major compounds of the most toxic EO (X. emarginata) with the binding pocket of the enzyme Acetylcholinesterase, a molecular target related to toxicity in models of Artemia salina. The chemical composition of the EO of D. echinophora was characterized by β-phellandrene (39.12%), sabinene (17.08%) and terpinolene (11.17%). Spathulenol (22.22%), caryophyllene oxide (12.21%), humulene epoxide II (11.86%) and allo-aromadendrene epoxide (10.20%) were the major constituents of the EO from D. riparia. Spathulenol (5.65%) and caryophyllene oxide (5.63%) were the major compounds of the EO from X. emarginata. The EO of X. frutescens was characterized by α-pinene (20.84%) and byciclogermacrene (7.85%). The results of the radical scavenger DPPH assays ranged from 15.87 to 69.38% and the highest percentage of inhibition was observed for the EO of X. emarginata, while for ABTS radical scavenging, the antioxidant capacity of EOs varied from 14.61 to 63.67%, and the highest percentage of inhibition was observed for the EO of X. frutescens. The EOs obtained from D. echinophora, X. emarginata and X. frutescens showed high toxicity, while the EO of D. riparia was non-toxic. Because the EO of X. emarginata is the most toxic, we evaluated how its major constituents were able to interact with the Acetylcholinesterase enzyme. The docking results show that the compounds are able to bind to the binding pocket through non-covalent interactions with the residues of the binding pocket. The species X. emarginata and X. frutescens are the most promising sources of antioxidant compounds; in addition, the results obtained for preliminary cytotoxicity of the EOs of these species may also indicate a potential biological activity.

## 1. Introduction

Essential oils (EOs) are complex mixtures of substances formed in the secondary metabolism of plants [1,2], and the substances present in EOs are intended to protect plants against pests, herbivores, fungi and bacteria [3]. Among these substances, sesquiterpenes, monoterpenes, aldehydes, alcohols, esters, and ketones stand out [4,5,6,7,8]. In aromatic species belonging to the *Annonaceae* family, compounds belonging to the class of mono and sesquiterpenoids have been identified as predominant [9,10]. Due to the strong demand for pure natural ingredients in various fields, EOs have been widely used all over the world for various applications in industrials sectors, such as food, pharmaceuticals and cosmetics production [11].

The antioxidant activity of EOs is a property of great interest because the EOs may preserve foods, cosmetics, perfumes and other products from the toxic effects of oxidants. Moreover, the ability of EOs to scavenge free radicals may play an important role in prevention of some diseases such as brain dysfunction, cancer, heart disease and immune system decline. Increasing evidence has suggested that these diseases may result from cellular damage caused by free radicals [12,13,14]. Furthermore, the *Artemia salina* Leach assay is a preliminary toxicity test that screens a large number of biosynthesized compounds from plant secondary metabolism and can quickly indicate the potential biological activity of EOs [15]. In general, authors report that the molecular target in toxicity tests with *A. salina* is acetylcholinesterase, so it is important to investigate the interaction mechanisms using in silico studies [16,17].

*Annonaceae* has numerous species that produce EOs. This family consists of 2106 species and more than 130 genera concentrated in the tropics. Around 900 species are neotropical, 450 are Afrotropical and the other species are Indomalayan [18]. In the Amazon region it is estimated that there are approximately 268 species [19]. The biological activities described for the EOs of these species include antioxidant [20,21,22] and cytotoxic activities [23]. Considering the large number of species of *Annonaceae* occurring in the Amazon region, there are still few studies investigating the chemical composition and the biological activities of the EOs of these species. In this paper, the chemical composition and the antioxidant and cytotoxic properties of the EOs obtained from the *Annonaceae* species collected in the State of Pará-Brazil (*Duguetia echinophora* R.E.Fr., *D. riparia* Huber, *Xylopia emarginata* Mart. and *X. frutescens* Aubl) were evaluated. We also studied the interaction of the major compounds of the most toxic EO with the binding pocket of the enzyme Acetylcholinesterase. 

It is worth mentioning that there is still no literature available on the biological properties of the EOs from *D. echinophora*, *D. riparia* or *X. emarginata* nor on the chemical composition of the EO of the species *D. echinophora*. The chemical composition of EOs from *D. riparia*, *X. emarginata* and *X. frutescens* has been evaluated and is characterized by mono and sesquiterpenes [24,25,26]. The EO from *X. frutescens* showed interesting anticancer [26] and repellent activities [27]. In folk medicine, this species is known in Brazil as “embira”, “semente-de-embira”, ‘‘embira-vermelha’’ and ‘‘pau carne”, and is widely used to treat flu, digestive problems, rheumatism, halitosis, tooth decay and as a bladder stimulant [26,28,29].

The present work provides new information related to the antioxidant potential of EOs from the species *D. echinophora, D. riparia, X. emarginata* and *X. frutescens* for use in areas such as food conservation. In addition, we investigate preliminary toxicity that provides important information related to the application of these EOs in potential biological activities.

## 2. Materials and Methods

### 2.1. Botanical Material

The leaves of *Annonaceae* species were collected in the municipality of Magalhães Barata (State of Pará, Amazon region, Brazil) in March 2018 (00°47′51.6″ S; 047°33′38.4″ samples were identified by Jorge Oliveira, a parataxonomist from the Museu Paraense Emílio Goeldi (MPEG), Belém, Pará, Brazil. The voucher specimens were deposited at the Herbarium of MPEG under the registration codes MG-237446 for *D. riparia*, MG-237477 for *D. echinophora*, MG-237444 for *X. frutescens* and MG-237449 for *X. emarginata*. 

### 2.2. Preparation of Botanical Material and Extraction of Essential Oils

The leaves of *Annonaceae* species were dried in an air-circulation oven for five days at 35 °C and then crushed in a knife mill (Tecnal, model TE-631/3, Piracicaba, São Paulo, Brazil). The moisture content was analyzed using a moisture analyzer (Marte, model ID50, São Paulo, Brazil). The EOs were extracted from the leaves of *Annonaceae* species by hydrodistillation in a glass modified Clevenger-type apparatus [30,31], using 150 g of plant material for each experiment. Hydrodistillations were carried out for 3 h at 100 °C. The obtained EOs were dried over anhydrous sodium sulfate and stored in a freezer at −10 °C. The yields of EOs (%) were calculated based on plant dry weight and expressed in mL/100 g of dried material.

### 2.3. Analysis of Chemical Profile of Essential Oil

The phytochemical profiles of the EOs were analyzed using chromatography/mass spectrometry (GC/MS) using a Shimadzu QP Plus 2010 GC-MS (Kyoto, Japan) following protocols reported earlier by our research group [32,33]. The retention index was calculated for all volatile constituents using a homologous series of *n*-alkanes (C_8_-C_40_, Sigma-Aldrich, St. Louis, MO, USA) according Van den Dool and Kratz [34], and the compounds were identified by comparing their mass spectrum and retention index with the data from the libraries [35].

### 2.4. ABTS•+ Radical Scavenging Assay 

The ABTS•+ assay was performed according to the methodology adapted from Miller et al. [36], and modified by Re et al. [37]. ABTS•+ was prepared using 7 mM ABTS•+ and 140 mM of potassium persulfate incubated at room temperature without light for 16 h. The solution was then diluted with phosphate-buffered saline until it reached an absorbance of 0.700 ± 0.02 at 734 nm. To measure the antioxidant capacity, 2.97 mL of the ABTS•+ solution was transferred to the cuvette, and the absorbance at 734 nm was determined using a Biospectro SP 22 spectrophotometer. Then, 0.03 mL of the sample was added to the cuvette containing the ABTS•+ radical, and after 5 min, the second reading was performed. The data were expressed as percent inhibition.

### 2.5. DPPH• Radical Scavenging Assay

The test was carried out according to the method proposed by Blois et al. [38]. To measure the antioxidant capacity, initially, the absorbance of DPPH• 0.1 mM diluted in ethanol was determined. Subsequently, 0.6 mL of DPPH• solution, 0.35 mL of distilled water, and 0.05 mL of the sample were mixed and placed in a water bath at 37 °C for 30 min. Thereafter, the absorbances were determined in a spectrophotometer at 517 nm. The data were expressed as percent inhibition.

### 2.6. Preliminary Toxicity

The toxicity of the essential oils was tested against larvae of the microcrustacean *Artemia salina* leach (brine shrimp). The eggs of *A. salina* (25 mg) were incubated at room temperature (27–30 °C) in an aquarium with artificial salt water composed of a mixture of 46 g of NaCl, 22 g of MgCl_2_.6H_2_O, 8 g of Na_2_SO_4_, 2.6 g of CaCl_2_.6H_2_O, and 1.4 g of KCl in 2.0 L of distilled water. The pH was adjusted to the 8.0–9.0 range using Na_2_CO_3_ to avoid the risk of larvae death by lowering the pH during incubation. After 24 h of egg hatching, oil solutions were prepared at concentrations of 100, 50, 25, 10, 5 and 1 µg·mL^−1^ using brine as vehicle and 5% dimethyl sulfoxide as diluent. Ten larvae of *A. salina* were placed in each tube containing the solution, and the mortality rate of the larvae after 24 h was calculated. The mean lethal concentration (LC_50_) was estimated using the Probitos statistical method. All the experiments were performed in triplicate using same protocols as described by Rebelo et al. [39].

### 2.7. In silico analysis

To carry out the in silico study, the molecules spathulenol and caryophyllene oxide (the major constituents present in the EO of *Xylopia emarginata*) were constructed using GaussView 5.5 software [40,41]. Their molecular structures were optimized with B3LYP/6-31G* [42,43] with Gaussian 09 [44]. We used the molecular method to evaluate the compounds interaction mode with Acetylcholinesterase (AChE). For this we used the Molegro Virtual Docker (MVD) 5.5 [45,46,47], and the crystal structure used as a molecular target can be found in the Protein Data Bank using the ID: 4M0E [48]. The MolDock Score (GRID) scoring function was used with a Grid resolution of 0.30 Å and 5 Å radius encompassing the entire connection cavity. The MolDock SE algorithm was used with the following parameter settings: number of runs equal to 10, maximum of 1500 interactions, and maximum population size equal to 50. The maximum evaluation of 300 steps with a neighbor distance factor equal to 1 and energy threshold equal to 100 was used during the molecular docking simulation.

### 2.8. Multivariate Analysis

The multivariate analysis was performed using the Minitab 17^®^ software (free version number 17, Minitab Inc., State College, PA, USA). The chemical constituents of the EOs from the leaves of *D. echinophora*, *D. riparia*, *X. emarginata* and *X. frutescens*, (≥3%), were set as the experimental variables, thus forming a matrix of 4 (samples) × 23 (variables) according to the literature [15,32,33]. 

## 3. Results and discussion

### 3.1. Chemical Composition

The EOs yields from the leaves of the Annonaceae species were 1.76, 0.08, 0.27 and 1.50% for *D. echinophora*, *D. riparia*, *X. emarginata* and *X. frutescens*, respectively. The yield found in this study for the EO of *D. riparia* was close to those found in studies with other species of the *Duguetia* genus (0.1–0.6%) [24]. The EOs yields found for the *Xylopia* species were also very close to those found in others studies [25,26]. The yields and EOs compositions of the species are shown in Table 1.

The chemical compositions of the EOs of *D. echinophora*, *D. riparia*, *X. emarginata* and *X. frutescens* were characterized by GC-MS, and a total of 22, 19, 59 and 62 components were identified, representing 96.40, 82.06, 81.69 and 95.08% of the total EOs for each species, respectively. The hydrocarbon monoterpenes compounds represented the most abundant class in the EOs of *D. echinophora* (78.99%) and *X. frutescens* (62.53%), and the oxygenated sesquiterpenes class characterized the EO of *D. riparia* (71.76%). The compounds β-Phellandrene (39.12%), sabinene (17.08%) and terpinolene (11.17%) were dominant in the *D. echinophora* EO, while spathulenol (22.22%), caryophyllene oxide (12.21%), humulene epoxide II (11.86%) and *allo*-aromadendrene epoxide (10.20%) were the major constituents of the *D. riparia* EO. The EO of *X. emarginata* was characterized by spathulenol (5.65%) and caryophyllene oxide (5.63%), and *X. frutescens* EO was characterized by α-pinene (20.84%) and byciclogermacrene (7.85%). Ion chromatograms are available in the Supplementary Material.

According to data previous published, the chemical compositions of the EOs of *Annonaceae* species occurring in Brazil are predominantly characterized by substances belonging to the class of mono and sesquiterpenes, and among these compounds, the most abundant are β-elemene, α-pinene, β-pinene limonene, bicyclogermacrene, (E)-caryophyllene, caryophyllene oxide, spathulenol, and germacrene D, [9]. 

Previous reports have investigated the chemical composition of the EOs from the *Annonaceae* species described in this work (*D. riparia*, *X. emarginata* and *X. frutescens*). The leaves and fine stems EO of *D. riparia*, also collected in State of Pará-Brazil, showed spathulenol (46.5%), caryophyllene oxide (28.9%) and α-pinene (6.1%) as their main compounds [24], and quantitative differences were observed for the constituents spathulenol and caryophyllene oxide in relation to the *D. riparia* EO described in the present work. The EO from the leaves of *X. emarginata*, collected in Caxiuanã National Forest, Melgaço, State of Pará-Brazil, showed a high percentage of sesquiterpene spathulenol (73.0%) [11], whereas in the present work, this constituent was obtained at a low percentage (5.65%) [25]. The EO from the leaves of *X. frutescens*, collected in Municipality of Capela, Sergipe State, Brazil, had as its major compounds (E)-caryophyllene (31.48%), bicyclogermacrene (15.13%), germacrene D (9.66%), δ-cadinene (5.44%), viridiflorene (5.09%) and α-copaene (4.35%) [26], while the EO from the leaves of the specimen collected in the city of Itabaiana, Sergipe-Brazil, had as its major constituents bicyclogermacrene (23.23%), (E)-caryophyllene (17.24%), β-elemene (6.35%) and (E)-β-ocimene (5.23%) [27]. 

The chemical composition of EOs can be strongly influenced by several factors, including season, climate, geography, age, genotype, organ, development periods, collection place and even extraction method, etc. [49,50,51]. Figueiredo and collaborators evaluated the influence of seasonal variation on the EO of *Eugenia patrisii* Vahl (*Myrtaceae*) and verified a potential correlation between the content of the main constituents of the essential oil and climatic parameters (temperature, insolation and humidity rate) [52]. The EOs of *Flos Chrysanthemi indici*, an important medicinal and aromatic plant in China, were obtained by different extraction techniques, hydrodistillation (HD), steam distillation (SD), solvent-free microwave extraction (SFME) and supercritical fluid extraction (SFE), and the authors found that the EO yield, chemical composition and bioactivities varied according to the extraction method used [53]. Some *Annonaceae* species have shown qualitative and quantitative variability in their EO compositions according to different collection sites. The EOs from the leaves of *Annona vepretorum* Mart. collected in the State of Sergipe, Brazil, showed bicyclogermacrene, spathulenol and α-phellandrene as the major constituents [54], while another specimen collected in the State of Pernambuco, Brazil, showed α-pinene, limonene, spathulenol and caryophyllene oxide as the compounds with higher percentage [55]. The compounds α-selinene, aristolochene, (*E*)-caryophyllene and (*E*)-calamenene were identified as the major constituents of EO from leaves of a specimen of *Duguetia lanceolata* collected in the state of Minas Gerais, Brazil [23], while another specimen collected in the State of São Paulo, Brazil, had as its main constituents of the EO the compounds *trans*-muurola-4(14),5-diene, β-bisabolene, 3,4,5-trimethoxy-styrene and 2,4,5-trimethoxy-styrene [56]. 

### 3.2. Multivariate Analyses

**Figure 1:**
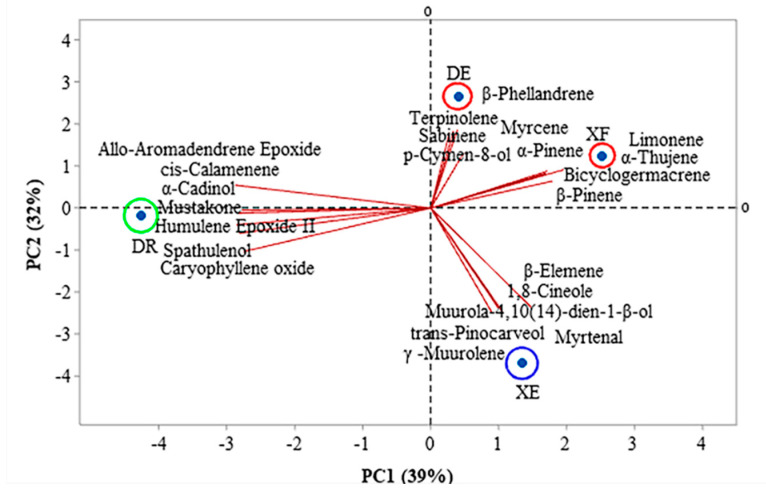
Biplot (principal component analysis) for the chemical analysis of the essential oils from *Annonaceae* species. DE: *Duguetia echinophora*; DR: *Duguetia riparia*; XE: *Xylopia emarginata*; XF: *Xylopia frutescens*.

**Figure 2:**
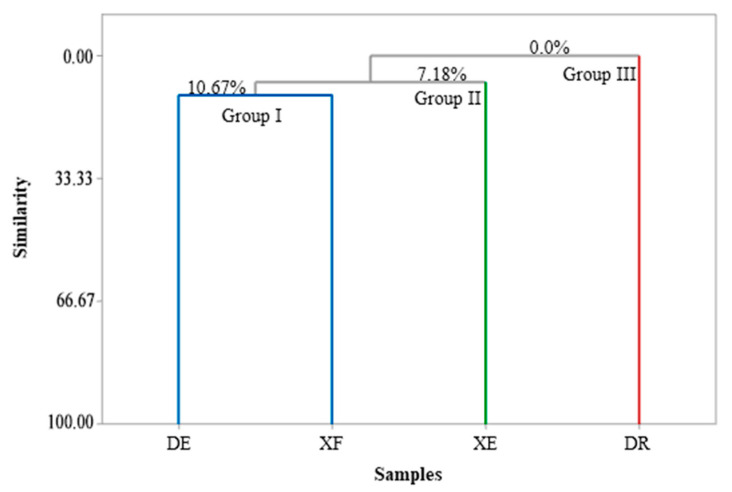
Dendrogram presenting the relational similarity of the chemical composition of the essential oils from *Annonaceae* species. DE: *Duguetia echinophora*; DR: *Duguetia riparia*; XE: *Xylopia emarginata*; XF: *Xylopia frutescens*.

Figure 1 and Figure 2 show the correlations between the classes of compounds identified in the different samples according to the multivariate analyses, principal component analysis (PCA) and hierarchical cluster analysis (HCA), respectively. PC1 and PC2 represent the principal components (PC), which contained 39.0% and 32.0% of the variables, respectively, and accounted for 71.0% of the variance in the analyzed data. In the HCA analysis, tree groups were observed that show the similarity between the identified classes. Group I, including the samples of EOs from *D. echinophora* and *X. frutescens* showed a similarity of 10.67% (Figure 2) and comprised the compounds β-phellandrene, *p*-cymen-8-ol, bicyclogermacrene, terpinolene, α-pinene, sabinene, myrcene, limonene, β-pinene and α-thujene (Figure 1). Groups II and III contained only one sample each and comprised β-elemene, 1,8-cineol, muurola-4,10(14)-dien-1-β-ol, trans-pinocarveol, myrtenal and γ-muurolene (*X. emarginata* EO) and cis-calamenene, α-cadinol, mustakone, *allo*-aromadendrene epoxide, humulene epoxide II, spathulenol and caryophyllene oxide (*D. riparia* EO), with similarities of 7.18% and 0%, respectively (Figure 2). 

### 3.3. Antioxidant Capacity 

**Figure 3:**
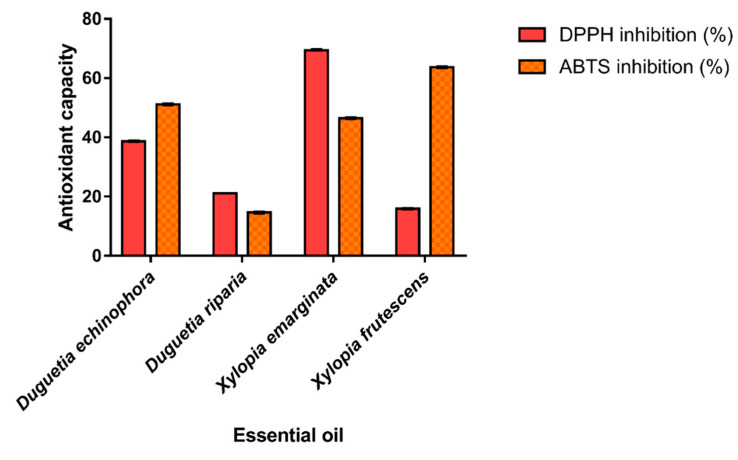
ABTS•+ and DPPH• radical scavenging assay and Trolox equivalent antioxidant capacity of essential oils. Values are expressed as mean and standard deviation (*n* = 3) of Trolox equivalent antioxidant capacity.

The antioxidant potential of the EOs from *Annonaceae* species was evaluated based on their ability to scavenge stable free DPPH• (2,2-diphenyl-1-picrylhydrazyl) and ABTS•+ (2,2′-Azino-bis (3-ethylbenzothiazoline-6-sulfonic acid) radicals; the results are shown in Figure 3. The results of the DPPH assays ranged from 15.87 to 69.38% and the highest percentage of inhibition was observed for the EO of *X. emarginata*, characterized by spathulenol (5.65%) and caryophyllene oxide (5.63%). For ABTS radical scavenging, the antioxidant capacity of EOs ranged from 14.61 to 63.67%. The species *X. frutescens* showed the higher antioxidant capacity by the ABTS•+ assay. This may be due to the presence of α-Pinene (20.84%) and β-Pinene (25.95%), the major components present in this EO. Possibly, the antioxidant activity of the *X. emarginata* EO can also be attributed to its main components which are described as antioxidants [57,58]. The high free radical scavenging effect of this sample may be related to the fact that the combination of the numerous organic chemical constituents present in EOs have a synergistic effect, increasing the biological activity or, conversely, an antagonistic effect [59]. In addition, bioactive compounds belonging to the monoterpenoid class have antioxidant activity, as reported in the literature [60].

Other studies investigating EOs of the *Duguetia* and *Xylopia* genera identified antioxidant effects. The EO of *Xylopia sericea* A. St.-Hil. showed significant antioxidant activity using DPPH (IC_50_ 49.1 μg·mL^−1^), β-carotene/linoleic acid bleaching (IC_50_ 6.9 μg·mL^−1^), TAC (IC_50_ 78.2 μg·mL^−1^) and TBARS (IC_50_ 80.0 μg·mL^−1^) methods [20]. The EO of *Duguetia lanceolata* St. Hil. branches showed antioxidant effects using a DPPH assay (EC_50_ 159.4 μg·mL^−1^), Fe^+3^ reduction (EC_50_ 187.8 μg·mL^−1^) and inhibition of lipid peroxidation (41.5%); the authors suggest that caryophyllene oxide is one of the active compounds found in this EO [21].

### 3.4. Cytotoxic Activity of Essential Oils

The toxicity of the EOs from *Annonaceae* species was measured in terms of LC_50_ (lethal concentration) with two negative control groups (control 1:10 nauplii and artificial sea-water with DMSO 0.1%; control 2: 10 nauplii and artificial seawater) and one positive control (K_2_Cr_2_O_7_, 50 µg·mL^−1^). The values are shown in Table 2. Values of LC50 < 80 µg·mL^−1^ are considered highly toxic [15,61,62]; values of LC_50_ within the range 80 to 250 µg·mL^−1^ are moderately toxic; and LC_50_> 250 µg·mL^−1^ are considered as low toxicity or non-toxic [63]. The EOs of *D. echinophora*, *X. emarginata* and *X. frutescens* showed high toxicity, whereas the EO of *D. riparia* showed low toxicity or was non-toxic. The major compounds from the EOs of *X. emarginata* (spathulenol and caryophyllene oxide) [64,65], *D. echinophora* (β-phellandrene and terpinolene) [66,67] and *X. frutescens* (α-pinene and byciclogermacrene) [68,69] showed cytotoxic effects and these results indicate that the cytotoxic potential observed for the EOs tested may be related to the presence of these secondary metabolites.

Toxicity tests in *A. Salina* performed with *Coriandrum sativum* L. (*Apiaceae*) showed an LC_50_ value of 23 µg/mL^−1^ [70], which is similar to those obtained in the present work for *D. echinophora* and X. *emarginata* EOs. Oliva and coworkers evaluated toxicity of the EOs from some medicinal plants, and the results showed a decreasing activity in the brine assay of *Aloysia polystachia* (*Verbenaceae*) (LC_50_ 6459 µg·mL^−1^), *Aloysia triphylla (Verbenaceae*) (LC_50_ 1279 µg·mL^−1^), *Minthostachys verticillata* (*Myrtaceae*) (LC_50_ 1848 µg·mL^−1^), and *Schinus poligamus* (*Anacardiaceae*) (LC_50_ 1179 µg·mL^−1^), that were considered nontoxic [71], Other authors have also reported the toxicity of essential oils from a variety of plants [17,72,73].

The essential oils of *Duguetia* species have been studied by using the *A. salina* bioassay. The EOs from the leaves, underground heartwood and underground stem bark of *Duguetia furfuracea* (A. St. -Hil.) Saff. showed potent activity against *A. salina* larvae (LC50 6.01, 7.79 and 9.98 μg·mL^−1^, respectively) and the leaf EO from *D. lanceolata* also showed potent activity against the same larvae (LC_50_ 0.89 μg·mL^−1^) [23]. In another study, the EOs of *D. lanceolata* showed toxicity against *A. salina* with LC_50_ values of 49.0 μg·mL^−1^ (2 h of hydrodistillation extraction) and 60.7 μg·mL^−1^ (4h of hydrodistillation extraction) [74].

### 3.5. Molecular Docking

**Figure 4:**
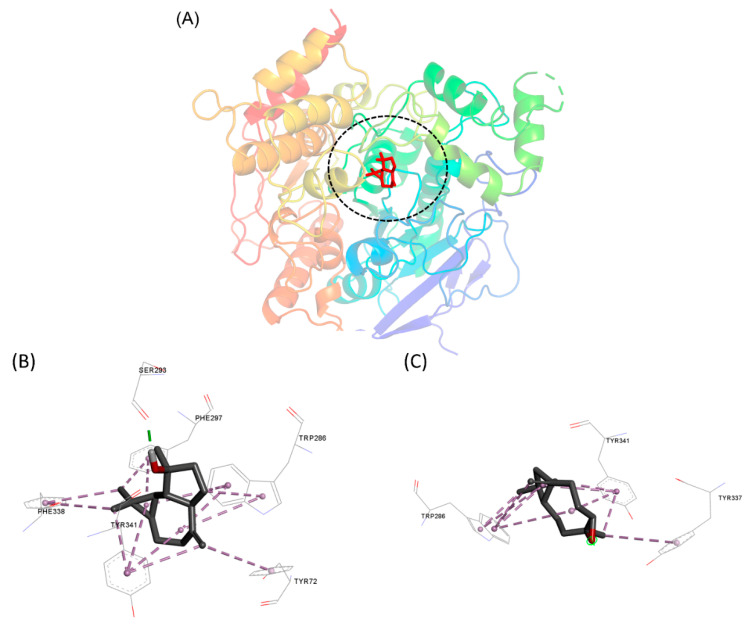
(**A**) Binding pocket of interaction of compounds with AChE. Molecular interactions established by (**B**) spathulenol and (**C**) caryophyllene oxide with the AChE active site.

Molecular modeling approaches have been used to investigate how natural compounds interact with molecular targets of pharmacological interest [75,76,77,78]. One of the tools used has been molecular docking, which can provide insights into how these compounds interact with the binding pocket of proteins. Here, we use this approach to assess how the major compounds of the EO from *X. emarginata* interact with the AChE active site, as this target is closely related to the toxicity mechanism observed in the *A. salina* assays [79,80]. Spathulenol formed hydrophobic interactions with various residues such as Ser293, Phe297, Trp286, Tyr72, Tyr341, and Phe338. A hydrogen bond was established with Ser293. Caryophyllene oxide established pi-alkyl hydrophobic interactions with Trp286, Tyr341 and Tyr337 (Figure 4). The interaction between spathulenol and caryophyllene with the active site of AChE has already been described [58] and this could be the likely mechanism responsible for the cytotoxicity of the EO from *X. emarginata*. 

## 4. Conclusions

The present study presents new insights concerning the chemical composition, antioxidant activity and preliminary toxicity of some *Annonaceae* EOs. Essential oils obtained from *D. echinophora, X. emarginata* and *X. frutescens* showed high toxicity, compared with EO obtained from *D. riparia,* which showed low toxicity or was non-toxic. The cytotoxicity test against *A. salina* can be considered as a good preliminary assessment of bioactive compounds, and may indicate a potential biological activity. The docking results elucidated the interaction mode of the major compounds of *X. emarginata* EO, spathulenol and caryophyllene, with the active site of the enzyme Acetylcholinesterase. The greatest capacities to scavenge DPPH and ABTS radicals were found in the essential oils of *X. emarginata* and *X. frutescens*, respectively, and the main constituents of the EO of this species may play the main role in the observed antioxidant capacity; however, the impact of less abundant constituents also should be considered.

## Figures and Tables

**Table 1:** Yield and chemical compositions of the *Annonaceae* species essential oils.

				**DE**	**DR**	**XE**	**XF**
Essential Oil Yield (%)				1.76	0.08	0.27	1.50
**R_T_**	**RI**_L_	**RI_C_**	Constituents (%)				
5.19	801	798	Hexanal			0.95	
6.95	844	845	Hex-(3*E*)-enol			1.35	
7.25	863	857	Hexanol			0.85	
8.30	924	925	α-Thujene				4.89
11.17	932	932	α-Pinene	4.14	1.31	3.14	20.84
11.23	946	948	Camphene			2.72	
11.98	969	974	Sabinene	17.08			
12.32	974	974	β-Pinene			2.01	25.95
12.57	988	991	Myrcene	3.61			
13.09	1002	1002	α-Phellandrene	1.27			1.73
13.56	1008	1011	δ-3-Carene	0.95			
13.89	1014	1016	α-Terpinene				0.85
14.02	1020	1022	*p*-Cymene	0.65		0.54	0.44
14.32	1024	1027	Limonene				3.00
14.79	1025	1029	β-Phellandrene	39.12			2.60
14.90	1026	1031	1,8-Cineole			3.36	1.00
14.99	1032	1043	(*Z*)-β-Ocimene				0.44
15.02	1054	1055	γ-Terpinene	1.00			1.40
15.13	1065	1068	*cis*-Hydrate sabinene				0.17
15.74	1086	1084	Terpinolene	11.17			0.39
15.87	1095	1099	Linalool			0.30	1.74
16.07	1112	1118	*trans*-Thujone				0.09
16.15	1114	1119	*endo*-Fenchol			0.33	
16.48	1118	1123	*cis*-*p*-Ment-2-en-1-ol				0.08
16.89	1122	1126	α-Campholenal			0.38	0.14
17.32	1135	1140	*trans*-Pinocarveol			4.46	0.36
17.54	1137	1149	*cis*-Verbenol			0.49	0.15
17.76	1154	1156	Sabina ketone			0.27	
17.94	1160	1162	Pinocarvone			2.35	0.16
17.98	1166	1168	*p*-Mentha-1,5-dien-8-ol			1.26	0.11
18.03	1167	1169	Umbellulone				0.04
18.09	1174	1180	Terpinen-4-ol	1.16			1.06
18.51	1179	1186	*p*-Cymen-8-ol	3.36		0.71	
18.74	1186	1194	α -Terpineol				0.97
18.82	1195	1196	Myrtenal			3.24	
18.91	1204	1207	Verbenone			1.62	0.1
19.13	1215	1218	*trans*-Carveol			0.33	
19.22	1239	1243	Carvone			0.23	
19.38	1249	1248	Geraniol			0.39	
19.53	1335	1335	δ-Elemene			2.32	4.41
19.68	1345	1345	α-Cubebene			0.74	0.08
19.95	1373	1367	α-Ylangene			1.35	
20.37	1374	1368	Isoledene				0.02
20.90	1374	1374	α-Copaene	0.25		1.07	0.25
21.56	1379	1378	Geranyl acetate				1.38
22.02	1387	1381	β-Bourbonene			0.93	
22.95	1389	1389	β-Elemene	0.74	0.49	3.10	0.54
23.68	1409	1405	α-Gurjunene			0.11	0.06
23.82	1417	1422	(*E*)-Caryophyllene	2.98	1.56	0.93	0.03
24.17	1419	1416	β-Ylangene				0.72
25.04	1434	1429	γ-Elemene		0.19	0.39	
25.26	1439	1439	Aromadendrene			0.75	0.39
26.13	1442	1442	6,9-Guaiadiene			0.06	
26.58	1451	1450	*trans*-Muurola-3,5-diene			0.38	
26.81	1452	1452	α-Humulene	0.73	1.40	0.35	0.10
27.05	1458	1456	*allo*-Aromadendrene	0.11			
27.19	1464	1465	(*E*)-9-*epi*-caryophyllene				0.26
27.81	1471	1470	Dauca-5,8-diene			0.29	
27.98	1478	1484	γ -Muurolene			3.06	
28.10	1484	1492	Germacrene D	1.24	1.34	1.08	3.26
28.18	1489	1487	β-Selinene			1.61	
28.29	1493	1494	*epi*-Cubebol			0.91	
28.33	1495	1490	γ-Amorphene			0.67	
28.52	1496	1489	Viridiflorene				0.56
28.61	1500	1497	Bicyclogermacrene	0.21			7.85
28.91	1500	1498	α-Muurolene			0.95	
29.03	1513	1513	γ-Cadinene			2.67	0.13
29.17	1514	1513	Cubebol		0.68		0.05
29.57	1522	1520	δ-Cadinene			1.61	0.38
29.98	1528	1520	*cis*-Calamenene	2.06	4.01	0.48	
30.06	1533	1531	*trans*-Cadina-1,4-diene			0.16	0.01
30.24	1533	1534	10-*epi*-Cubebol				0.09
30.39	1537	1536	α-Cadinene			0.24	
30.58	1539	1540	α-Copaen-11-ol				0.04
30.67	1544	1540	α-Calacorene			1.47	
30.83	1548	1548	Elemol				0.06
31.57	1564	1561	β-Calacorene			0.65	
31.97	1577	1579	Spathulenol	1.87	22.22	5.65	2.18
32.28	1582	1583	Caryophyllene oxide	2.49	12.21	5.63	0.18
32.51	1590	1589	Globulol				1.10
32.62	1592	1593	Viridiflorol		0.61		0.54
32.76	1595	1594	Cubeban-11-ol				0.23
32.85	1596	1596	Fokienol		2.48		
32.92	1600	1604	Rosifoliol				0.23
33.09	1602	1601	Ledol				0.10
33.25	1608	1609	Humulene Epoxide II	0.21	11.86	1.41	
33.81	1630	1630	Muurola-4,10(14)-dien-1-β-ol			4.70	
34.18	1638	1643	*epi*-α-Cadinol				0.09
34.36	1639	1657	*Allo*-Aromadendrene Epoxide		10.20		0.02
34.45	1639	1661	Caryophylla-4(12),8(13)-dien-5-α-ol		1.36		
34.51	1640	1664	*epi*-α-Muurolol				0.12
34.71	1644	1669	α-Muurolol			0.83	
34.84	1645	1672	Cubenol		2.57	0.68	
34.89	1648	1678	*cis*-Guaia-3,9-dien-11-ol				0.54
34.93	1652	1681	α-Cadinol		3.45		0.30
35.49	1668	1684	*trans*-Calamenen-10-ol			0.30	
35.62	1668	1692	14-Hydroxy-9-*epi*-(*E*)-caryophyllene			1.00	
35.94	1676	1694	Mustakone		3.36		
36.28	1685	1695	Germacra-4(15),5,10(14)-trien-1-α-ol		0.76	1.40	0.04
39.41	1767	1768	14-oxi-α-Muurolene			0.48	
59.61	2400	2408	Tetracosane				0.02
62.38	2500	2512	Pentacosane				0.02
Monoterpenes hydrocarbon Oxygenated monoterpenes Sesquiterpenes hydrocarbon Oxygenated sesquiterpenes Others class				78.99	1.80	8.41	62.53
				4.52	0	19.45	6.17
				8.32	8.50	27.42	19.05
				4.57	71.76	22.99	5.03
				-	-	3.42	2.30
Total				96.4	82.06	81.69	95.08

RT: Retention Time; RI_C_ = Calculated retention index; RI_L_ = Literature retention index; DE: *Duguetia echinophora*; DR: *Duguetia riparia*; XE: *Xylopia emarginata*; XF: *Xylopia frutescens*.

**Table 2:** LC_50_ concentrations of the essential oils using *Artemia salina* assay.

Essential Oil	LC_50_ (µg·mL^−1^)
*Duguetia echinophora*	28.00 ± 0.30
*Duguetia riparia*	310.80 ± 0.70
*Xylopia emarginata*	26.72 ± 0.17
*Xylopia frutescens*	54.36 ± 0.20
Positive control (K_2_Cr_2_O_7_)	50.00 ± 0.00

Values are expressed as mean and standard deviation (*n* = 3).

## Data Availability

The data are contained within the article and supplementary materials.

## Supplementary Materials

The following supporting information can be downloaded at: https://www.mdpi.com/article/10.3390/antiox11091709/s1, Figure S1: Ions-chromatogram relating to the chemical profile of essential oils from different species of *Annonaceae Xylopia*.
