# Supplementary material for: Phytochemical Profile, Antioxidant Potential and Toxicity Evaluation of the Essential Oils from Duguetia and Xylopia Species (Annonaceae) from the Brazilian Amazon
Source: Antioxidants (Basel). 2022 Aug 30;11(9):1709. doi: 10.3390/antiox11091709 (PMC9495368; doi:10.3390/antiox11091709)

Supplementary material. S1.

Phytochemical Profile, Antioxidant Potential and Toxicity Evaluation of the Essential Oils from *Duguetia* and *Xylopia* Species (*Annonaceae*) from Brazilian Amazon

Márcia Moraes Cascaes[1], Ângelo Antônio Barbosa de Moraes[2], Jorddy Neves Cruz[3], Celeste de Jesus Pereira Franco[2], Renan Campos e Silva[1], Lidiane Diniz do Nascimento[2], Oberdan Oliveira Ferreira[4], Tainá Oliveira dos Anjos[5], Mozaniel Santana de Oliveira[2*], Giselle Maria Skelding Pinheiro Guilhon[1], Eloisa Helena de Aguiar Andrade[1,2,4]

[1]Programa de Pós-Graduação em Química, Universidade Federal do Pará, Rua Augusto Corrêa S/N, Guamá, Belém 66075-900, PA, Brazil. giselle@ufpa.br (G. M. S. P. G), renan.c.silva@outlook.com (R.C.S)

[2]Laboratório Adolpho Ducke-Coordenação de Botânica, Museu Paraense Emílio Goeldi, Av. Perimetral, 1901, Terra Firme, Belém 66077-830, PA, Brazil. eloisa@museu-goeldi.br (E.H.A.A.). angeloquimica17@gmail.com (Â.A.B.M). lidianenascimento@museu-goeldi.br (L.D.N.), celeste.frango12@gmail.com (C.J.P.F.)

[3]Laboratory of Functional and Structural Biology, Institute of Biological Sciences, Universidade Federal do Pará, Rua Augusto Corrêa S/N, Guamá, Belém 66075-900, Pará, Brazil. jorddynevescruz@gmail.com (J.N.C)

[4]Programa de Pós-Graduação em Biodiversidade e Biotecnologia-Rede Bionorte, Universidade Federal do Pará, Rua Augusto Corrêa S/N, Guamá, Belém 66075-900, PA, Brazil. oberdan@museu-goeldi.br (O.O.F.)

[5]Programa de Pós-graduação em Ciências Biológicas-Botânica Tropical, Museu Paraense Emilio Goeldi/Universidade Federal Rural da Amazônia, Av. Perimetral, 1901, Terra Firme, Belém 66077-830, PA, Brazil. tainadosanjoscb@gmail.com (T.O.A)

* Correspondence: cascaesmm@gmail.com; Tel.: +55-91-982024161 (M.M.C); mozaniel.oliveira@yahoo.com.br or mozanieloliveira@museu-goeldi.br; tel +55 91 988647823 (M.S.O).

Supplementary material. S1.

Figure S1. Ions-chromatogram relating to the chemical profile of essential oils from different species of *Annonaceae.*

Xylopia emarginata.

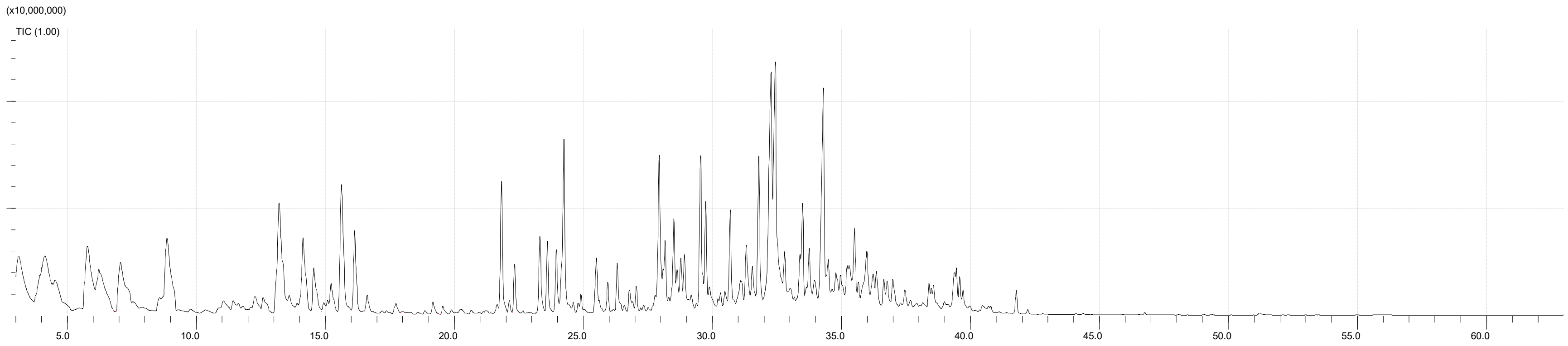

Xylopia frutescens

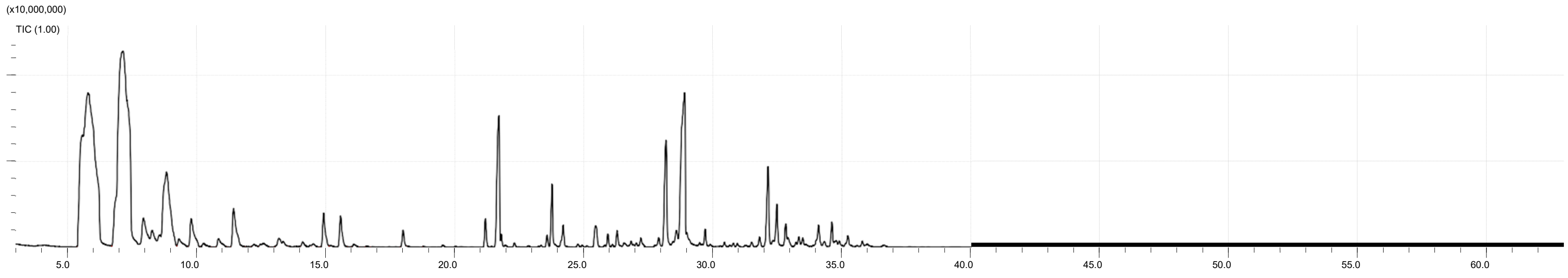

Dugietia riparia

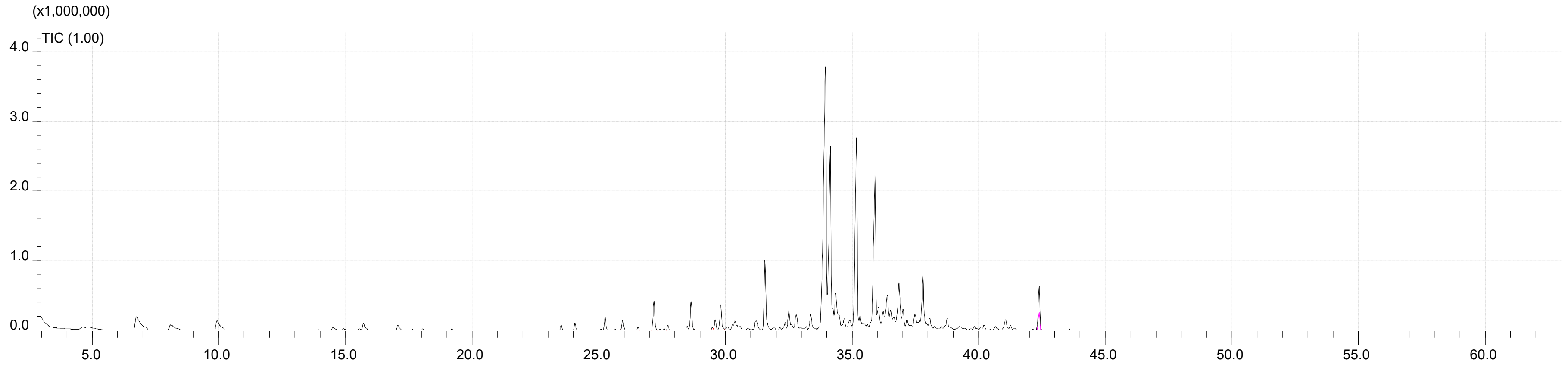

Duguetia echinophora

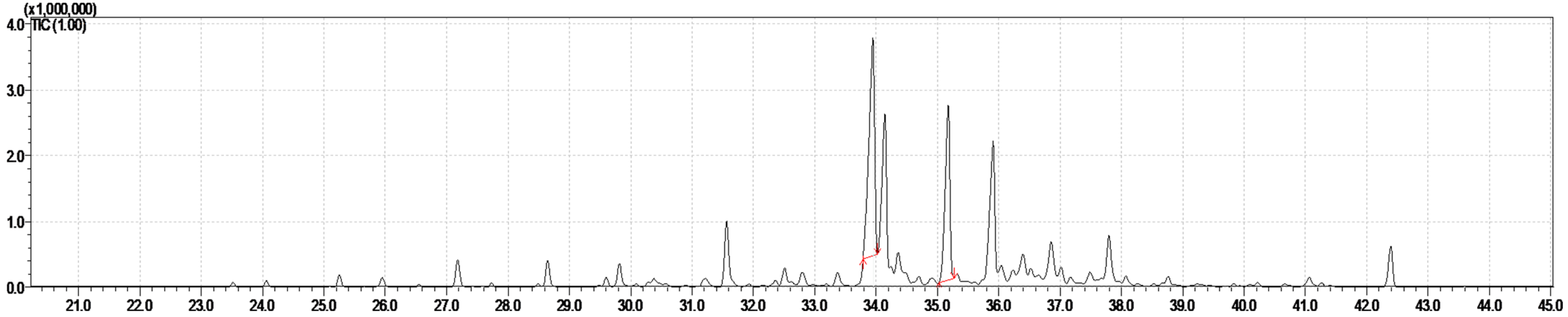

Supplement: Supplementary file 1 [file antioxidants-11-01709-s001.zip › antioxidants-1864164-supplementary.pdf]
